# Coronary Atherosclerosis in Master Athletes: Current Knowledge and Future Challenges

**DOI:** 10.3390/jpm16030172

**Published:** 2026-03-23

**Authors:** Ioannis Boutsikos, Themis Gkraikou, Richard Saad, Alexandros Kasiakogias, Ioannis Patrikios, Argyrios Ntalianis, Dimitrios Chatzis

**Affiliations:** 1 Department of Therapeutics, Alexandra University Hospital, National and Kapodistrian University of Athens, 11528 Athens, Greece; arg_nt@yahoo.gr; 2 Department of Internal Medicine, Mediterraneo Hospital, 16675 Athens, Greece; drthemisgkraikou@gmail.com; 3 Department of Cardiology, Asklepieion General Hospital, 16673 Athens, Greece; richardsaad@hotmail.com; 4 1st Department of Cardiology, University of Athens, 11527 Athens, Greece; akasiakogias@gmail.com; 5 School of Medicine, European University Cyprus, Nicosia 2404, Cyprus; i.patrikios@euc.ac.cy (I.P.); dimitrioschatzis@yahoo.com (D.C.)

**Keywords:** sports cardiology, coronary calcium score, endurance exercise, atherosclerosis, elite athletes

## Abstract

Coronary atherosclerosis in master athletes represents a paradox: despite the well-established cardiovascular benefits of regular exercise, highly trained endurance athletes show a higher prevalence of coronary plaques than their non-athletic peers. The mechanisms behind this finding are multifactorial, involving sustained high shear stress on the vascular wall, exercise-induced inflammatory activation, altered calcium homeostasis, and interactions between genetic predisposition and sport-specific lifestyle factors. Although athletes tend to exhibit predominantly calcified—potentially more stable—plaques, recent studies highlight that mixed and non-calcified lesions are also present, particularly among lifelong endurance athletes, raising questions about their true long-term risk. Clinically, traditional risk scores often underestimate risk in this population, making multimodal assessment with tools such as coronary calcium scoring and coronary CT angiography essential. This review synthesizes the current knowledge on mechanisms, clinical implications, diagnostic strategies, and prevention of coronary atherosclerosis in athletes, while underscoring key gaps that future research must address.

## 1. Introduction

Coronary artery disease (CAD) is a progressive disorder affecting a substantial part of the general population, running subclinically at its initial stages while becoming more likely to be clinically overt over time [1]. Coronary artery plaque formation, the cornerstone of CAD is typically related to a sedentary lifestyle, poor dietary habits and smoking. While physical inactivity is an additional factor for CAD [2], it is known that participation in high-intensity competitive sport activities increases the level of myocardial stress [3].

In the current era, it is common for master athletes defined as people older than 35 or 40 years to engage with vigorous and intense training programs. The dose–response relationship, though, between long-term intensive endurance exercise and coronary heart disease is a matter of debate. Early studies suggested that regular endurance sport activity exerts protective effects on ischemic heart disease [4]. Yet recent studies have detected an increased prevalence of CAD and atherosclerotic plaques amongst highly trained athletes compared with healthy non-athletes [5]. The literature has focused on coronary atherosclerosis in athletes mainly because exercise-related death in athletes over 35 years old is mostly associated with CAD [6]. As a matter of fact, understanding the unique mechanisms and clinical manifestations of CAD in athletes is crucial for early detection and appropriate management strategies.

In the present review we aim to investigate and shed light upon the challenging concept of CAD and atherosclerotic plaque formation and progression in highly trained master athletes.

## 2. Potential Etiologies

Atherosclerotic plaque formation and progression in master athletes may be multifactorial regarding its etiology, and the precise underlying mechanisms are not fully understood. Potential etiologies are listed below and summarized in Table 1.

### 2.1. Shear Stress on Vascular Walls Due to Intense Physical Activity

Exercise causes hyperdynamic blood flow in the coronary arteries, especially at sites of turbulent flow as bifurcations, potentially precipitating endothelial damage. Oscillatory flow dynamics lead to endothelial dysfunction and the formation of reactive oxygen species (ROS), leading to atheromatic formation and progression [7]. Moreover, the process of coronary endothelium repairment results in elevated calcium depositions on the affected areas and ultimately in the development of atherosclerotic plaques [8]. Furthermore, during intense endurance training, increased mechanical pressure may lead to the destabilization or rupture of the plaque, leading to thrombus and microemboli [9].

There are also data supporting that intensive endurance exercise may in fact accelerate the progression of pre-existing CAD rather than leading to de novo atherosclerotic plaques [10]. Interestingly, it has been reported that endurance exercise is associated with CAD progression among master runners with already established disease and with an increase in non-calcified plaques, which are more prone to rupture [11].

### 2.2. Immune System Mobilization Due to Exercise

Recent research suggests that inflammation plays a crucial role in the development of atherosclerotic plaques beyond the traditional cardiovascular risk factors. More specifically, pro-inflammatory cytokines are released as a consequence of high-intensity exercise [12]. Macrophages that engulf low-density lipoprotein (LDL) transition to their foam-like appearance and induce the production of chemokines and inflammatory mediators. The inflammasome that is formed marks the instability of the atherosclerotic plaque and finally the progression of CAD. Moreover, it seems that intensive endurance training increases not only myocardial oxygen consumption but also the generation of reactive oxygen species (ROS) such as superoxide and hydrogen peroxide [13].

### 2.3. Induced Calcium Homeostasis

Parathyroid hormone (PTH) is a key factor for coronary atherosclerosis, as it is one of the main regulators of calcium homeostasis and is hypothesized to be linked to cardiovascular disease. Exercise stimulates the secretion of PTH, whose levels are proportionally correlated with training duration and intensity [13,14]. In particular, high-intensity endurance exercise has been associated with elevated circulating PTH concentrations. Elevated PTH, calcium and phosphate homeostasis are predictive of arterial calcification, as noted by several studies with coronary computed tomography [15].

### 2.4. Dietary Habits, Food and Performance Boosters

Dietary habits impact the development and progression of CAD. Highly competent athletes report a higher energy intake compared with the general population, with higher total amounts of carbohydrates and protein intake [16]. Moreover, performance boosters and energy drinks often used by athletic individuals are of undetermined significance regarding cardiovascular health [17].

### 2.5. Genetics

Genetics is a key factor for the development of atherosclerosis, and twin studies have shown a strong genetic predisposition towards calcified plaque volumes [18]. There is no evidence indicating genetic differences amongst athletes and the general population concerning the genetic substrate of atherosclerosis. However, a UK Biobank study suggested that cardiorespiratory fitness, but not physical activity volumes, could attenuate CAD risk [19]. Notably though, the plausible epigenetic modification of exercise on intermediate- and high-risk populations is an ongoing research topic and could probably lead to a better understanding of atherosclerotic plaque dynamics and their potential modifications from external stimuli [20].

**Figure 1:**
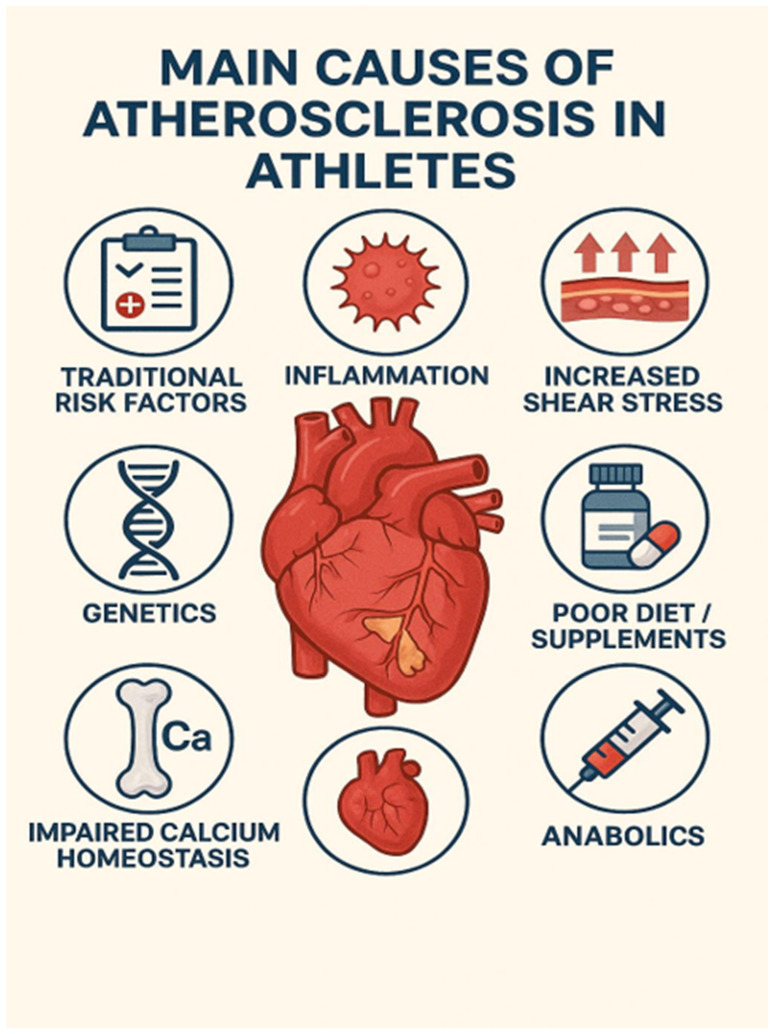
Schematic representation of potential etiologies of coronary atherosclerosis in athletes.

The main causes of coronary atherosclerosis in athletes are depicted in Figure 1.

### 2.6. Clinical Implications

It is of great importance to address the topic of CAD in master athletes. The concern and question many researchers and physicians are trying to address is whether athletes are protected from plaque formation/progression and associated rupture. Evidence arising from research and certain examples of Olympic champions indicate that athletes are not protected from plaque instability. There is a basic understanding of the evolution process from stable to unstable plaques, which is used as a compass to understand and try to predict vulnerability. This is a dynamic process with different underlying pathophysiological mechanisms, and adding the potential role of intense endurance exercise to the equation further perplexes the whole situation [3,21]. In recent years, there have been enormous strides towards understanding the details pertaining to atherosclerotic plaques, mainly due to the use of modern imaging modalities, such as coronary CT, which have revealed important insights, expanding our knowledge base in order to better understand the concept of atherosclerosis in master athletes [22].

In the former, the presence of coronary plaques highlights both potential benefits and drawbacks of high-intensity training. While exercise is generally protective, athletes are not immune to the development of atherosclerotic coronary disease, including unstable plaques that may precipitate cardiac events. Nonetheless, the health benefits of exercise are undeniable. If physical activity could be packaged as a medication, it would represent the most powerful therapy available, benefiting not only the heart but virtually every organ system from head to toe. Even low-to-moderate levels of exercise have consistently been shown to reduce cardiovascular risk and improve long-term outcomes [23,24].

Extensive research on exercise physiology has demonstrated that coronary circulation undergoes significant adaptive changes at both structural and functional levels. Structurally, exercise promotes the development of new vessels, enhancing myocardial perfusion. Functionally, it induces beneficial adaptations in vascular motor control that optimize oxygen delivery to meet the increased myocardial demands of physical activity. These adaptive mechanisms become considerably more complex in the presence of coronary stenosis, where flow regulation is further challenged [25]

Several mechanisms have been proposed to explain exercise-induced vascular adaptations, including collateral vessel formation through angiogenesis, as well as improvements in endothelial function. Yet exercise may also create imbalances in endothelial regulation under certain conditions.

A key question in master athletes is whether exercise can contribute to atherosclerosis regression. If so, what aspects of the plaque are modified by physical activity? Addressing these questions requires the detailed assessment of plaque composition and its structural changes in response to exercise.

One of the pivotal investigations in this field was the German marathon study, published in 2008. This study evaluated nearly 100 apparently healthy middle-aged male marathon runners and compared them with age- and risk factor-matched controls. Coronary calcium scores—a well-established marker of cardiovascular risk when >100—were elevated in 36% of marathon runners versus 22% of controls. These findings challenged the assumption that endurance training confers protection against coronary atherosclerosis, instead demonstrating that a high coronary calcium burden remained the strongest prognostic indicator. Long-term follow-up confirmed that marathon runners with elevated calcium scores had significantly worse event-free survival, despite all having completed at least five marathons [26].

A decade later, the landmark MARC-1 study extended these observations in more than 300 asymptomatic middle-aged male cyclists. A substantial proportion demonstrated coronary calcification, and several exhibited severe disease (interpretation of these findings requires caution). Although participants were self-identified as “healthy”, many displayed cardiovascular risk factors, including elevated blood pressure, a high prevalence of former smoking, diabetes, hypercholesterolemia, family history of CAD, and frequent use of lipid-lowering therapy [27]. Table 2 summarizes the main studies on coronary atherosclerosis in athletes.

Further analyses revealed a clear relationship between lifelong exercise volume and coronary calcification; greater training volume was associated with more advanced coronary calcification [31]. Interestingly though, athletes with plaques were more likely to have predominantly calcified lesions compared with less active counterparts. Similarly, findings from the London group confirmed that male master endurance athletes exhibited a higher burden of calcified plaque relative to controls [32,33].

This distinction may be clinically relevant because unstable plaques are characterized by features such as thin fibrous caps, large lipid cores, and spotty calcium; thus, the predominance of calcified plaques in athletes may reflect a more stable phenotype, potentially less prone to rupture and acute coronary events [34]. Accordingly, it needs to be clarified that plaque burden and plaque stability constitute two different entities and, in the case of male master athletes undergoing high-volume endurance exercise, it seems that plaque calcification represents a rather protective effect (as in the case of statins) and not a sign of accelerated disease progression.

While this is an idea that is intuitively easy to understand, our knowledge about coronary calcification in athletes has yet again been challenged by the results of a study from Belgium. This study included a large cohort of controls, late-onset endurance athletes and lifelong endurance athletes, all middle-aged males, who had undergone cardiac CT [30]. The study revealed that there was a clear relationship between exposure to intense endurance exercise and coronary plaque development, as previously demonstrated by relevant studies. It has also been shown that lifelong endurance master athletes had the greater burden of non-calcified and mixed atherosclerotic plaques compared with the other study participants. It should be noted, though, that in the study, controls had nearly no risk factors in comparison with the endurance athletes [30].

In addition, evidence suggests that there is a whole spectrum of different factors that could influence plaque formation in athletes [3,33]. While a number of these are non-modifiable, there are also a number of factors that point towards sports specific interactions and sports specific risk factors that could be influencing the presence of plaque and coronary calcification [31].

Such findings raise the question and speculation that a combination and interaction of different exercise-driven and exercise-influenced risk factors lead towards the development of plaques in athletes and active individuals.

A recent experimental study by Guasch and colleagues investigated the vascular effects of varying exercise intensities in mice subjected to sedentary, moderate-, and high-intensity training protocols. The findings in the group of high-intensity training revealed maladaptive vascular remodeling, particularly within the tunica media, where fibrosis and smooth muscle stiffening were observed. These changes impaired vascular dilation and constriction balance, potentially offsetting some of the beneficial effects typically associated with exercise [35].

Complementing these mechanistic insights, a randomized clinical trial from Norway examined lipid core formation in individuals with coronary plaques—an uncommon study design in sports cardiology. The trial demonstrated that while high-intensity training did not significantly alter overall lipid core burden, higher VO_2_ peak levels were associated with reductions in lipid content, suggesting improved plaque stability [28].

Beyond intermediate outcomes, the Cooper Center longitudinal study provided large-scale evidence in more than 20,000 individuals followed for over a decade. The study demonstrated that the relative risk associated with coronary calcification increased progressively with higher physical activity levels, particularly beyond 1500 and 3000 MET min per week. These findings highlight the complex relationship between exercise volume, coronary calcification, and long-term cardiovascular outcomes [36].

Moreover, the timing and frequency of exercise appear to influence risk. A recent meta-analysis of 12 case-crossover studies including nearly 20,000 patients showed that cardiac events were more likely to occur during exercise in individuals who were not habitual exercisers. In contrast, this risk was significantly lower among those who engaged in exercise almost daily [37].

This echoes a concept first articulated by Maron in 2000: the “double-edged sword” of exercise. While acute exertion may transiently increase the risk of sudden cardiac death in susceptible individuals with underlying coronary disease, habitual exercise provides substantial long-term protection. In summary, athletes derive marked cardiovascular benefit from regular physical activity, but they are not immune to adverse events. Exercise represents a complex interplay of protective and risk-enhancing mechanisms, particularly in relation to coronary plaque and calcification [6]. However, the results of the presented studies cannot be generalized to specific subsets such as female athletes, strength and power athletes.

### 2.7. Screening and Risk Stratification in Athletes

#### 2.7.1. Risk Assessment and Predictive Models

The identification of athletes at increased cardiovascular risk represents a major challenge in sports cardiology. Traditional risk scores, such as SCORE2 or the Framingham risk score, were developed in general populations and frequently underestimate the risk of master athletes because of their favorable cardiovascular profile and low prevalence of classical risk factors [38,39]. Despite apparently optimal metabolic indices, several studies have demonstrated a disproportionately high burden of coronary artery calcification in endurance athletes when compared with less-active cohort of similar age [5,29]. Importantly, the calcification pattern in athletes is often characterized by a predominance of calcified and mixed plaques rather than lipid-rich lesions, suggesting a different pathophysiological substrate that may carry a lower risk of rupture but still needs careful monitoring [40,41]. Table 3 summarizes the characteristics of calcified and non-calcified plaques.

Coronary artery calcium (CAC) scoring has emerged as a particularly strong prognostic marker in this setting. Progression of CAC is strongly associated with incidents of coronary events, even in individuals classified as low risk by conventional risk scores. In athletes, CAC measurement has been shown to reclassify a significant proportion into higher risk categories, thereby altering therapeutic management. The astronaut cardiovascular health and risk modification (AstroCHARM) model was developed to address these inadequacies by integrating traditional clinical risk factors with CAC and high-sensitivity C-reactive protein (hs-CRP) levels, thereby improving predictive accuracy in populations with unique physiological adaptations such as astronauts and endurance athletes [42].

#### 2.7.2. Diagnostic Modalities in Risk Stratification

A comprehensive diagnostic strategy is essential to distinguish physiological adaptations of the athlete’s heart from pathological findings. Resting ECG and echocardiography represent first-line investigations, allowing assessment of structural abnormalities and differentiation between adaptive remodeling and cardiomyopathy [9,10]. Maximal exercise stress testing remains an important tool, uncovering exertional ischemia or arrhythmia not apparent at rest and helping define safe thresholds for sports participation and/or return-to-play decisions [43].

CAC scoring has a central role in risk reclassification, particularly in master athletes, with higher scores correlating with future coronary events [43]. CCTA further enhances diagnostic accuracy by providing direct visualization of coronary stenosis severity and plaque morphology, distinguishing calcified from non-calcified lesions [41]. Ambulatory ECG monitoring is recommended in athletes with palpitations or syncope, permitting correlation of symptoms with arrhythmic events [29]. Collectively, these modalities permit an individualized assessment that integrates anatomy, ischemia, and arrhythmic potential.

### 2.8. ESC Guidelines on Athlete Screening

The 2020 ESC Guidelines on Sports Cardiology recommend a tailored approach to screening, according to age and level of risk [43]. In greater detail, for athletes younger than 35 years, the aim is the early identification of genetic predisposition such as congenital or inherited cardiac disorders such as hypertrophic cardiomyopathy, arrhythmogenic right ventricular cardiomyopathy, congenital coronary anomalies, and channelopathies. The baseline evaluation should include a detailed personal and family history, a thorough physical examination, and a 12-lead electrocardiogram (ECG). Upon abnormal ECG findings, further investigation should be performed with imaging such as echocardiography, cardiac MRI, or genetic testing [44].

**Figure 2:**
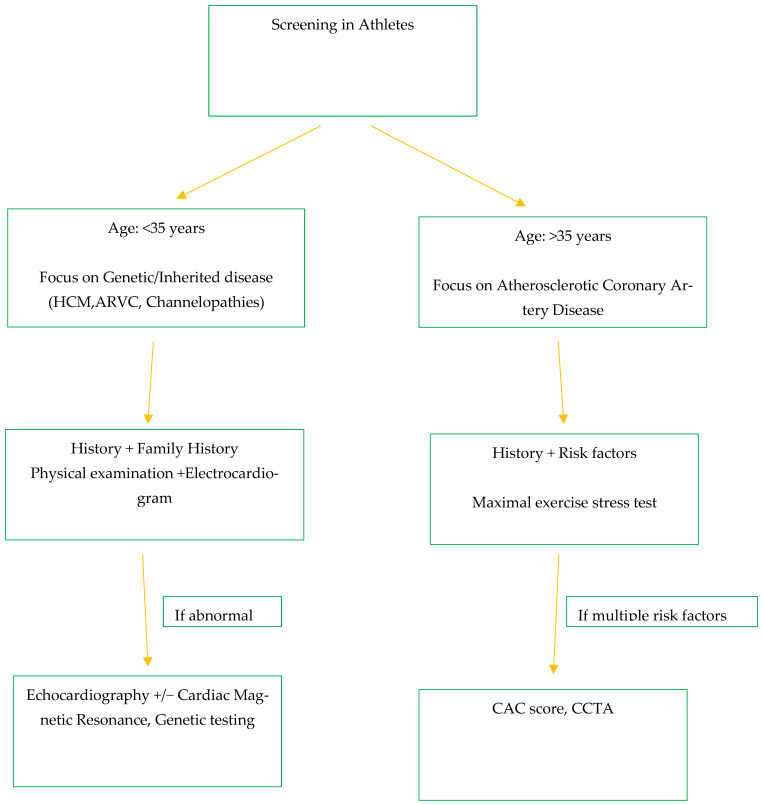
Algorithm flowchart for screening in athletes according to ESC guidelines from 2020. (HCM—hypertrophic cardiomyopathy, ARVC—arrhythmogenic right ventricular cardiomyopathy, CAC—coronary artery calcium score, CCTA—coronary computed tomography angiography.

In contrast, in athletes over 35 years of age, atherosclerotic CAD is the principal pathology of concern. In this group, conventional risk estimation tools are frequently inaccurate, particularly in highly trained individuals, as their professional exercise-induced adaptations may mask underlying subclinical disease [5,45]. The guidelines therefore recommend maximal exercise stress tests in symptomatic athletes or those with multiple risk factors, as well as the selective use of CAC scoring and coronary CT angiography (CCTA) for patients with a normal maximal stress test but multiple risk factors such as diabetes, family history of CAD, or previous risk assessment suggesting a high risk for CAD. These imaging modalities provide detailed information on coronary plaque burden and morphology, enabling more accurate risk stratification and personalized recommendations [34]. Figure 2 summarizes the aforementioned algorithm.

### 2.9. Management of Atherosclerotic Disease in Athletes

#### 2.9.1. ESC Guidance on Sports Participation

The 2020 ESC Guidelines on Sports Cardiology underline that participation decisions should not follow rigid thresholds but instead be made on an individualized basis [43]. Athletes with stable coronary artery disease, preserved systolic function, and no evidence of inducible ischemia may be permitted to resume low- or moderate-intensity competition once clinical stabilization and medical therapy have been achieved. In carefully selected low-risk cases, participation in more demanding activities can also be considered, provided that a comprehensive evaluation—including maximal exercise testing or, ideally, cardiopulmonary exercise testing (CPET)—confirms the absence of ischemia, arrhythmias, or abnormal hemodynamic responses [43]. In such circumstances, a gradual, supervised increase in training load is recommended, while restrictions remain particularly relevant for disciplines with high cardiopulmonary demands, such as endurance, mixed, and power sports, especially in older athletes [45]

Conversely, athletes with reduced ejection fraction, persistent symptoms despite optimal therapy, or high-risk features such as exercise-induced arrhythmias should generally be restricted to low-intensity skill-related sports or recreational activity, where the physiological burden is limited and the risk of adverse outcomes minimized [43,46].

#### 2.9.2. The American College of Cardiology Guidance on Sports Participation

The most recent American statement (AHA/ACC, 2025) represents a paradigm shift compared with earlier documents and the ESC guidelines. These differences are summarized in Table 4. Its central principle is shared decision making (SDM): participation in competitive sports for athletes with cardiovascular abnormalities is no longer based on absolute prohibitions, but rather on individualized risk assessment and a joint decision involving the athlete, the cardiologist with sports cardiology expertise, and when necessary, a multidisciplinary team.

The guidelines maintain a 14-point history and physical examination as the minimum screening tool, while they consider a 12-lead ECG as an option in asymptomatic athletes, provided that there is expertise in interpretation and a system for appropriate downstream testing. In contrast, there is no recommendation for universal ECG or imaging screening for all athletes. Importantly, any screening program should be coupled with an emergency action plan (EAP), training in CPR, and availability of AEDs at training and competition venues.

Special attention is given to master athletes (≥35 years), in whom CAD is the main concern. The guidelines recommend careful risk stratification, aggressive risk factor management, and the use of advanced imaging modalities (CAC, CCTA) where appropriate. Continuous monitoring and dynamic risk evaluation are emphasized, recognizing that risk evolves over time [47].

### 2.10. Preventive Strategies

Preventive care in athletes requires reconciling the paradox of increased coronary calcification with the protective effects of high fitness levels (Table 5). Statins remain the cornerstone of therapy, supported by strong evidence for LDL-C reduction and plaque stabilization. In athletes, concerns about statin-associated muscle symptoms may be mitigated by using hydrophilic formulations, alternate-day dosing, or adjuncts such as coenzyme Q10 [48]. The use of low-dose aspirin is considered selectively in those individuals with extensive CAC or high-risk plaque characteristics, while also evaluating bleeding risk [49].

Nutritional interventions are of paramount importance, with the Mediterranean diet shown to improve vascular health and support recovery from training [50]. Adequate protein intake, restriction of refined sugars, and an emphasis on fruits, vegetables, and omega-3 fatty acids provide additional benefit. Lifestyle measures, including smoking cessation, moderation of alcohol, stress reduction, and optimization of sleep hygiene, reinforce plaque stability and overall cardiovascular health [48].

Emerging evidence has highlighted the influence of the gut microbiome in mediating the cardiovascular benefits of exercise. Experimental studies suggest that endurance exercise can attenuate Western diet-induced atherosclerosis through microbiota-mediated mechanisms, reducing systemic inflammation and enhancing vascular protection [49]. Such findings underscore the multifaceted benefits of regular physical activity beyond traditional risk factor modification. 

### 2.11. Multifactorial Control

Management of coronary disease in athletes should address all modifiable risk factors in a coordinated manner. Lipid lowering remains central, with statins as first-line agents, supplemented by ezetimibe or PCSK9 inhibitors where targets are not achieved or intolerance develops [50]. Blood pressure control should be pursued with agents that preserve exercise capacity, such as ACE inhibitors or ARBs [43]. In athletes with diabetes or impaired glucose tolerance, SGLT2 inhibitors or GLP-1 receptor agonists may offer dual cardiovascular and metabolic benefits without impairing performance [47].

Comprehensive prevention extends to lifestyle modification, including nutritional optimization, smoking cessation, and psychosocial support. Serial monitoring with biomarkers (lipids, hs-CRP, HbA1c) and imaging modalities such as CAC or CCTA enables dynamic adjustment of therapy. Finally, multidisciplinary management involving cardiologists, sports physicians, nutritionists, physiologists, and psychologists provides a holistic framework for sustaining cardiovascular safety and athletic performance [51].

### 2.12. Sex Differences and Coronary Atherosclerosis in Female Athletes

Sex-related differences represent an important, yet insufficiently explored, modifier of coronary atherosclerosis in athletic populations. Most studies investigating coronary plaque burden in athletes have predominantly included male participants, thereby the applicability of their findings to women are limited. Emerging evidence, however, suggests that the relationship between exercise volume and subclinical coronary atherosclerosis differs substantially between sexes. A recent meta-analysis demonstrated that increasing exercise volume is associated with a higher burden of subclinical coronary atherosclerosis in men, whereas this association appears attenuated or absent in women, despite comparable or even superior levels of cardiorespiratory fitness [51].

These findings are supported by a dedicated imaging study of female master endurance athletes, which reported a low prevalence of coronary atherosclerosis, minimal coronary artery calcium burden, and predominantly non-obstructive disease when present [52]. Importantly, even among women with lifelong exposure to endurance exercise, the absolute prevalence of coronary plaques remained substantially lower than that reported in male cohorts. Observations from studies including mixed male–female athletic populations are consistent with these data, demonstrating lower plaque prevalence and burden in women compared with men when assessed by coronary computed tomography angiography, despite similar exposure to high-volume endurance training [30,53,54].

Collectively, available evidence suggests that female athletes may be less susceptible to the development of exercise-associated coronary atherosclerosis. Nevertheless, the limited number of female participants, heterogeneity of study designs, and lack of longitudinal data preclude definitive conclusions. These limitations highlight the need for prospective studies specifically designed to evaluate coronary plaque burden, composition, and clinical significance in female athletic populations across different ages and levels of exercise exposure.

## 3. Conclusions

Coronary atherosclerosis in athletes represents a paradoxical phenomenon that challenges the long-standing perception of exercise as uniformly protective against cardiovascular disease. Large observational cohorts have demonstrated that lifelong endurance training is associated with a greater prevalence of coronary plaques compared with less active peers, particularly calcified and mixed morphologies [5,34,42]. Importantly, although coronary artery calcium (CAC) burden is often higher in athletes, plaques tend to exhibit more stable features, with a lower proportion of vulnerable non-calcified lesions, which may partly explain the paradoxical coexistence of increased subclinical atherosclerosis with lower incidence of acute coronary events [27,31]. This paradox illustrates the intricate nature of vascular adaptations to prolonged intense exercise and emphasizes the importance of differentiating structural imaging findings from their actual prognostic relevance. From a clinical standpoint, the accurate identification of high-risk individuals remains paramount.

The relevant studies are rather observational, which does not guarantee the causal relationship between high-volume endurance exercise and atherosclerosis. Athletes represent the healthiest part of the population, but there are confounding factors like smoking habits, poor dietary habits, poor regulation of lipids and blood pressure. Overweight status should also be taken into consideration when it comes to correlating high-volume endurance exercise with the developing of atherosclerosis.

Traditional risk calculators, such as the Framingham risk score or SCORE, frequently underestimate cardiovascular risk in athletes. Consequently, multimodal evaluation—including coronary CT angiography, CAC scoring, cardiopulmonary exercise testing, and biochemical profiling—may provide greater precision in stratifying risk. However, it must be emphasized that the use of CAC and/or CCTA is not intended for everyone as it may be important concerns regarding cost-effectiveness, radiation exposure and other issues. We propose, in an effort to prevent unnecessary testing, that specific subgroups of elite athletes such as those with CAC > 100, positive family history of premature coronary artery disease, as well as former or current smokers may benefit the most from advanced imaging. The 2020 ESC Guidelines on Sports Cardiology recommend individualized decision making, allowing asymptomatic athletes with low-risk coronary profiles to safely participate in training, while advising restrictions in those with high-risk features such as left main or multivessel disease, impaired ejection fraction, or inducible ischemia [43].

Management should therefore be personalized, integrating preventive cardiology and sports medicine. Alongside conventional interventions—including lipid lowering, blood pressure control, and lifestyle optimization—tailored exercise prescriptions and shared decision making between athletes and clinicians are essential [22,43,47]. Experimental models further suggest that exercise confers additional cardio protection by modulating systemic inflammation, improving endothelial function, and even altering gut microbiota composition toward a less atherogenic environment [49]. These results suggest that, even in the presence of structural coronary atherosclerosis, regular physical activity continues to provide an overall protective effect on cardiovascular health. Current evidence on coronary atherosclerosis in athletes is mostly represented by male cohorts and cannot be directly extrapolated to female athletes. Emerging data suggest a lower plaque prevalence and a weaker association between high-volume endurance exercise and subclinical coronary atherosclerosis in women, emphasizing the need for sex-specific, prospective studies.

Future research should address critical gaps, particularly through multi-ethnic cohorts including women and under-represented populations, in order to further elucidate our knowledge regarding the potential association of high-volume endurance exercise and coronary atherosclerosis. Furthermore the integration of functional capacity measurements (e.g., VO_2_ max) with atherosclerosis plaque progression, together with the assessment of inflammatory and microbiota biomarkers may also enhance our current knowledge and enrich our insights regarding this rather complex potential association. Clarifying whether exercise-induced calcification represents a benign adaptive remodeling or an early marker of heightened risk will be central to advancing clinical care. Ultimately, bridging preventive cardiology, advanced imaging, and individualized sports medicine will be essential to balance the benefits of lifelong exercise with the nuanced risks of coronary atherosclerosis in athletes.

## Figures and Tables

**Table 1:** Key factors of coronary atherosclerosis in athletes.

Category	Key Factors
Non-Modifiable Risk Factors	Age (especially master athletes, >35–40), genetic predisposition (family history, high cholesterol, metabolic disorders)
Traditional Cardiovascular Risk Factors	Elevated LDL/low HDL, hypertension, diabetes or insulin resistance, smoking or second-hand smoke exposure, obesity/central adiposity (even with normal weight, fat distribution matters)
Exercise Volume and Intensity	Very high endurance training over many years, long sessions of high-intensity or prolonged exercise without adequate recovery, repetitive mechanical/hemodynamic stress on arteries (shear stress fluctuations)
Inflammation and Oxidative Stress	Repeated oxidative bursts during intense training, micro-trauma and local inflammatory responses, poor rest/recovery increasing chronic inflammation
Diet/Nutrition/Supplement Use	Diet high in saturated fats, trans fats, cholesterol; imbalanced antioxidant vs. pro-oxidant intake; supplements or substances that may negatively affect lipids, blood pressure, or endothelial function
Other Lifestyle and Environmental Factors	Poor sleep or sleep deprivation, exposure to pollutants/toxins, use of performance-enhancing substances with cardiac risks, psychological or physical stress
Plaque Composition and Nature in Athletes	More common to find calcified plaques vs. soft/fibro-lipid plaques (potentially less rupture-prone), mixed/non-calcified plaques are higher risk but appear less frequently in highly trained endurance athletes, presence of plaque does not always correlate with symptoms or high risk but interacts with other factors

**Table 2:** Key studies on coronary atherosclerosis in athletes (CAC—coronary artery calcium score).

Study	Population	Key Findings
German Marathon Study *(2008)* [26]	~100 male marathon runners vs. controls	36% of runners had high CAC score vs. 22% of controls; high calcium burden predicted worse outcomes
MARC-1 Study (*2016)* [27]	>300 middle-aged male cyclists	Significant coronary calcification; some had severe CAD despite low traditional risk
Cooper Center Longitudinal Study *(2019)* [28]	>20,000 individuals	High physical activity linked to higher CAC but improved overall survival
Vasaloppet Ski Race *(2013)* [29]	>50,000 cross-country skiers	Higher endurance exposure → increased AF risk
De Bosscher et al. *(2023)* [30]	Lifelong vs. late-onset endurance athletes vs. controls	Lifelong endurance athletes had more coronary plaques; no protective calcified phenotype

**Table 3:** Characteristics of calcified and non-calcified coronary plaques.

Characteristic	Calcified Coronary Plaques	Non-Calcified Coronary Plaques
Histopathological composition	Predominantly calcium phosphate (hydroxyapatite) deposits within a fibrotic or necrotic core	Lipid-rich necrotic core, fibrous tissue, proteoglycans; may include inflammatory cell infiltrates
Stage of atherosclerosis	Generally represent more advanced, chronic, or stabilized lesions	Often represent earlier or intermediate stages of atherosclerosis
Plaque stability	Usually considered more stable	Often considered less stable, especially lipid-rich plaques
Association with plaque rupture	Lower risk of rupture (except spotty or microcalcifications)	Higher risk of rupture, particularly in thin-cap fibroatheromas
Association with acute coronary syndromes (ACS)	Less commonly implicated directly in ACS	Strongly associated with ACS and myocardial infarction
Effect on coronary artery calcium score (CAC)	Contributes directly to CAC score	Not detected by CAC scoring
Detection by non-contrast CT	Readily detectable	Not detectable

**Table 4:** Differences in ESC and AHA/ACC guidelines concerning sports participation and master athletes.

	ESC 2020	AHA/ACC 2025
Central element	Focus on prevention of sudden cardiac death, often more restrictive	Shared decision making, individualized approach, fewer absolute prohibitions
Screening < 35 yrs	Target: genetic/inherited diseases. Baseline: history, exam, ECG; further imaging if abnormal	14-point history and physical examination; ECG “reasonable” if expertise available; no universal screening
Screening ≥ 35 yrs	Target: CAD. Stress testing, CAC/CCTA in selected cases	Emphasis on CAD in master athletes. Risk stratification; CAC/CCTA when appropriate
Sport classification	Categorized by static/dynamic load and intensity	Continuum approach considering actual demands of the sport
Re-evaluation	Not always explicitly highlighted	Dynamic assessment, continuous monitoring
Arrhythmias/cardiomyopathies/myocarditis	More conservative, often restrictive	Individualized, avoids blanket prohibitions
Emergency preparedness	Not a central element	Mandatory: emergency action plan, CPR training, AED availability

**Table 5:** Preventive strategies in athletes with coronary atherosclerosis.

Strategy	Benefits
Statins	LDL-C reduction, plaque stabilization; hydrophilic statins preferred in athletes
Aspirin (selective use)	Considered in extensive CAC or high-risk plaque; balance with bleeding risk
Lifestyle	Mediterranean diet, smoking cessation, stress reduction, adequate sleep
Nutritional optimization	High protein, reduced refined sugars, omega-3 fatty acids
Blood pressure control	ACE inhibitors/ARBs preferred (preserve exercise tolerance)
Diabetes management	SGLT2 inhibitors/GLP-1 agonists—metabolic and CV benefits
Microbiome effects	Exercise-induced gut flora changes reduces systemic inflammation

## Data Availability

No new data were created or analyzed in this study. Data sharing is not applicable to this article.
